# Case Report: Recurrent Variant c.298 TA in *CCN6* Gene Found in Progressive Pseudorheumatoid Dysplasia Patients From Patni Community of Gujarat: A Report of Three Cases

**DOI:** 10.3389/fgene.2021.724824

**Published:** 2021-09-28

**Authors:** Harsh Sheth, Jhanvi Shah, Aadhira Nair, Premal Naik, Jayesh Sheth

**Affiliations:** ^1^ FRIGE’s Institute of Human Genetics, Ahmedabad, India; ^2^ Rainbow Superspeciality Hospital and Children’s Orthopedic Centre, Ahmedabad, India

**Keywords:** progressive pseudorheumatoid dysplasia, recurrent variant, Patni, Gujarat, *CCN6*

## Abstract

Biallelic mutations in the *CCN6* gene are known to cause a rare genetic disorder—progressive pseudorheumatoid dysplasia (PPD). PPD is characterized by distinct joint deformities of interphalangeal joints, stiffness, gait disturbance, abnormal posture, and absence of inflammation, resulting in significant morbidity. The largest case series of PPD from India suggests c.233G>A and c.1010G>A to be the most common mutations in the *CCN6* gene, although the distribution of these variants among endogamous communities in India has not been carried out. We here report three cases of PPD from three independent families belonging to the Patni community of Gujarat, a community known to practice endogamy. All three cases had short stature, gait disturbance, scoliosis, and interphalangeal joint deformities. Analysis by whole-exome sequencing in the first case showed the presence of a previously known, homozygous, missense variant c.298T>A (p.Cys100Ser) in exon 3 of the *CCN6* gene in all cases. Due to all three families belonging to the same community, analysis by Sanger sequencing in the remaining two cases for the variant mentioned earlier showed both cases to be of homozygous mutant genotype. Unaffected family members, i.e., parents and siblings, were either heterozygous carriers or wildtype for the said variant. The present case series is the first report of a recurrent variant occurring across multiple PPD-affected individuals from unrelated families belonging to the same community from India.

## Introduction

Progressive pseudorheumatoid dysplasia (PPD; OMIM#208230), also known as spondyloepiphyseal tarda with progressive arthropathy or progressive pseudorheumatoid arthropathy of childhood, is a rare autosomal recessive disorder (Dalal et al., 2012). PPD patients are asymptomatic at birth but progressively develop joint deformities, specifically interphalangeal joints, stiffness, joint contractures, gait imbalance, scoliosis, and/or kyphosis, leading to abnormal posture and swelling in hips, knees, wrists, and fingers (Dalal et al., 2012; Bhavani et al., 2015). Short stature (<third centile), absence of inflammation, and pain are considerable phenotypes in these patients. The condition is part of a group of 42 genetic and clinically stratified groups of skeletal disorders (Chen et al., 2016).

PPD occurs due to missense and/or nonsense variants in the *CCN6* (Wnt1 lnducible signaling pathway 3) gene located on chromosome 6q22 (Hurvitz et al., 1999). CCN6 protein is a member of the connective tissue growth factor/cysteine-rich 61/nephroblastoma overexpressed (CCN) family of the extracellular matrix of proteins involved in the regulation of cell migration, adhesion, proliferation, differentiation, and survival (Hurvitz et al., 1999). CCN6 protein function has been reported to be in maintaining cartilage integrity, and abnormal protein function could lead to degradation and loss in articular cartilage (Wang et al., 2013).

The majority of the variants in the *CCN6* gene have been reported from the United States, China, and Middle-East countries. More recently, variant data from the largest series of PPD patients from 79 families from India were published, showcasing a series of novel mutations and reclassification of one variant (Dalal et al., 2012; Bhavani et al., 2015). Interestingly, five previously reported variants—c.156C>A, c.233G>A, c.383C>A, c.739_740delTG, and c.1010G>A—were observed in several patients across several families, with c.233G>A and c.1010G>A suggested to be specific to the Indian population (Bhavani et al., 2015).

Herein, we report three PPD cases from three self-reportedly unrelated families belonging to the Patni community of Gujarat. Furthermore, we present a novel observation of a previously known pathogenic variant c.298T>A to be present in all cases from a given community.

## Case Description

### Clinical Findings

Three probands from three self-reportedly unrelated families belonging to the Patni community of Gujarat in India came for genetic counseling.

#### Case 1

**FIGURE 1:**
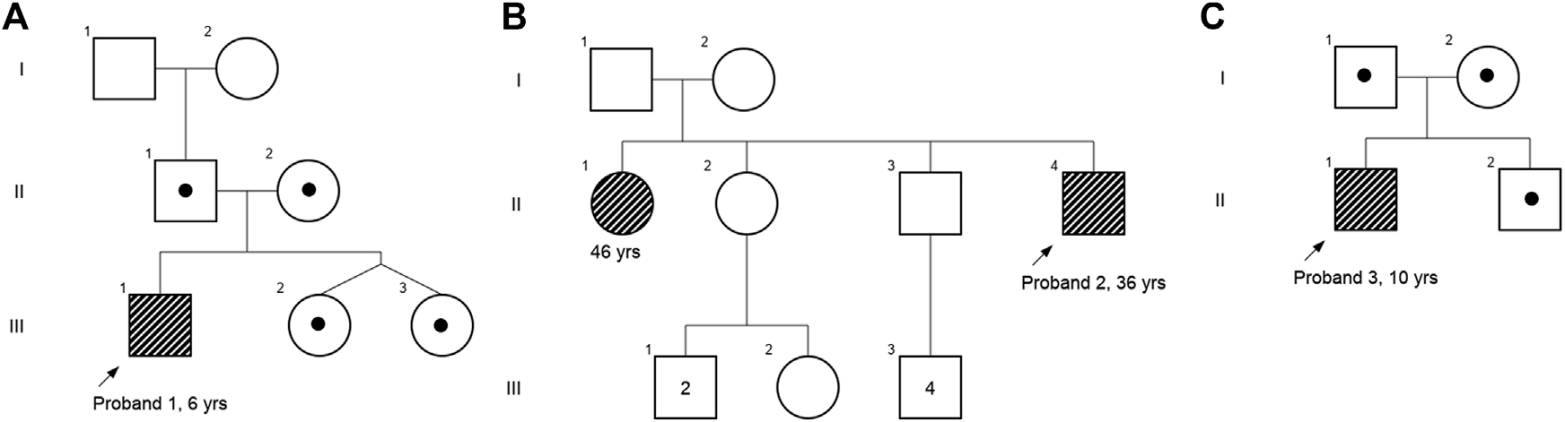
Pedigree chart of (**A**) case 1, (**B**) case 2, and (**C**) case 3. Affected individuals are shaded in gray strips, and carriers are depicted with a dot.

**FIGURE 2:**
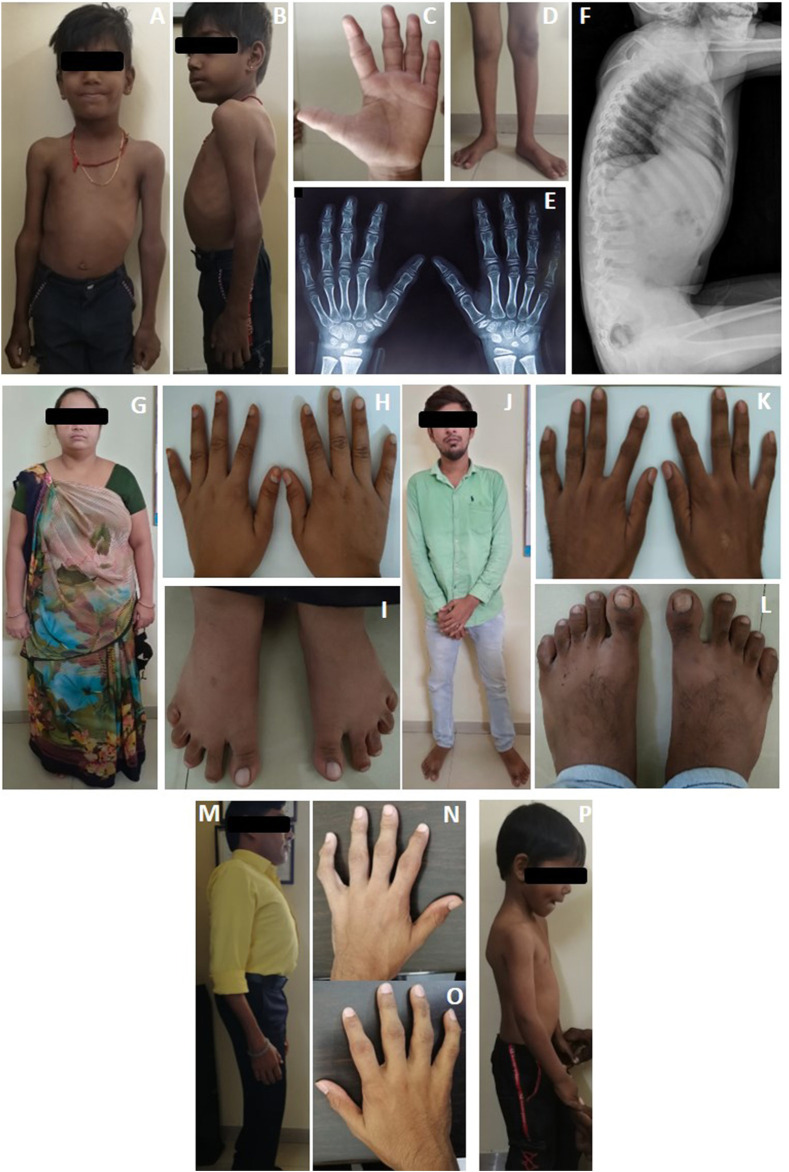
Clinical photographs and radiographs of proband's (**A–F**) case 1, (**G–H**) case 1’s mother, (**J–L**) case 1’s father, (**M–O**) case 2, and (**P**) Case 3. Case 1 is observed with (**A**) chest deformity and enlarged elbow joints; (**B**) scoliosis; (**C**) deformities of interphalangeal joints; (**D**) knocked knees; (**E**) radiograph anterior-posterior view, showing osteopenia; (**F**) anterior beaking of vertebrae. (**G–I**) Clinical picture of mother (carrier) of case 1; (**J–L**) clinical picture of father (carrier) of case 1. Case 2 is observed with **(M)** scoliosis and **(N,O)**clinodactyly with deformities of interphalangeal joints. Case 3 is observed with **(P)** mild scoliosis.

A 6-year-old male proband, born to a phenotypically normal and endogamous couple (Figure 1A), presented with prominent distal and proximal bilateral metaphysis of fingers, gait disturbance, mild scoliosis, anterior beaking of the chest, square vertebrae, and osteopenia (Figures 2A–F). He had a normal complete blood count and rheumatoid factor of 6.20 IU/ml (normal range: 0–20 IU/ml). Phenotypic analysis of the mother showed a normal clinical picture (Figures 2G–I). However, the father showed mild enlargement of interphalangeal joints of the hands (Figure 2J–L).

#### Case 2

A 36-year-old male proband, born to a phenotypically normal and endogamous couple (Figure 1B), presented with short stature, bilateral clinodactyly, gait disturbance, scoliosis, square vertebrae, enlarged chest, and inward knocked knees (Figures 2M–O). His 46-year-old female sibling was reported to have a similar set of phenotypes. Other family members were reported to be phenotypically normal.

#### Case 3

A 10-year-old male proband, born to a phenotypically normal and endogamous couple (Figure 1C), presented with short stature, mild scoliosis, reduced flexion of fingers with incomplete fist formation, and bilateral elbow terminal flexion restriction (Figure 2P).

### Molecular Findings

Genomic DNA was extracted from ethylenediaminetetraacetic acid-treated peripheral whole blood using the desalting method and quantified using QIAxpert (Qiagen, Germany). Whole-exome sequencing was carried out in case 1 only. Library preparation was carried out using the Agilent SureSelect Clinical Research Exome V2 capture kit (Agilent, United States) and sequenced on the Illumina HiSeq platform (Illumina, United States). Fastq files were aligned against the GRCh37/hg19 reference genome using BWA v0.7.17, and an average coverage of 118.48 × post-alignment was observed (Li and Durbin, 2009). Variants were called using GATK’s HaplotypeCaller v4.1 by following GATK’s best practices framework (Poplin et al., 2018). Variants were filtered and prioritized using human phenotype ontology-coded phenotype-driven Exomiser v12.1.0 tool (Smedley et al., 2015). A known pathogenic homozygous variant CCN6(NM_003880.4):c.298T>A (p.Cys100Ser) was observed in exon 3 of the *CCN6* gene at a depth of 80×.

**FIGURE 3:**
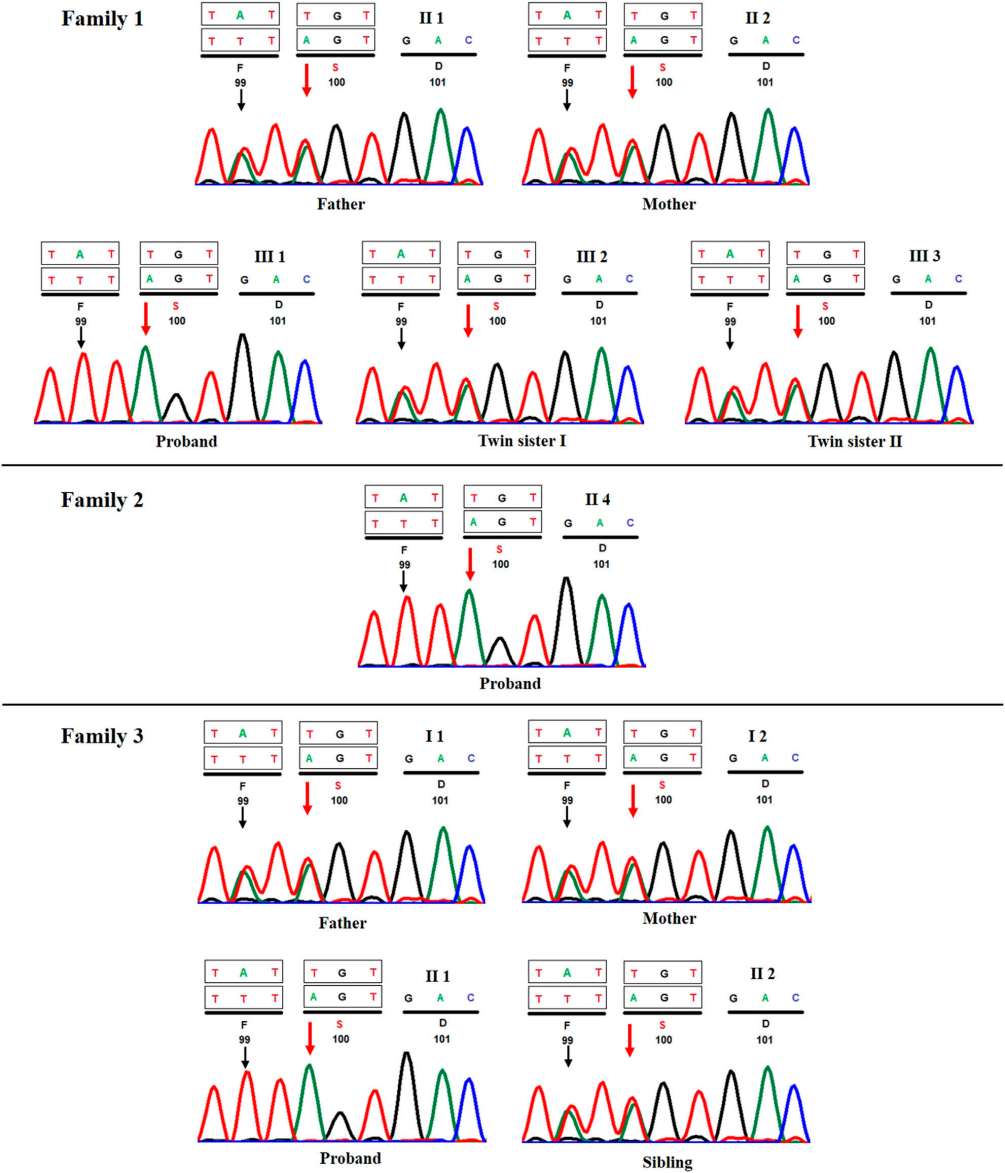
Sanger sequencing chromatograms of all three proband's and their respective first-degree relatives (**A**) case 1, (**B**) case 2, and (**C**) case 3. Red and black arrows show the position of c.298T>A and c.296A>T variants, respectively.

Because probands from all three families belonged to the same ethnic community, we prepared an *a priori* hypothesis that the variant detected in case 1 would likely be observed in cases 2 and 3 due to the prevalence of endogamous practices within the community. Therefore, Sanger sequencing of variant c.298T>A was carried out in cases 2 and 3 and their parents. Interestingly, we observed both cases 2 and 3 to be homozygous for the said variant, and their first-degree relatives (i.e., unaffected parents and/or siblings) were found to be heterozygous carriers (Figure 3).

## Discussion

PPD is a rare skeletal dysplasia with disruption of cartilage homeostasis and progressive joint stiffness in the absence of inflammation (Wynne-Davies et al., 1982). The disease is caused due to either dysfunction or loss of the CCN6 protein. Its prevalence has been estimated to be 1 per 1 million in the United Kingdom population. However, it is likely that the disease may be underdiagnosed due to overlapping clinical presentations with juvenile idiopathic arthritis (Hurvitz et al., 1999).

To date, approximately 210 families have been reported with an underlying genetic cause of PPD, with the most common occurrences in communities practicing endogamy/consanguinity (Progressive Pseudorheumat, 2021). The largest set of case series comprising 79 families has been published from India (Dalal et al., 2012; Bhavani et al., 2015). In total, Bhavani *et al.* reported 9 novel mutations and 17 mutations that have so far been observed only in the Indian population (Bhavani et al., 2015). Interestingly, the authors observed c.156C>A (p.Cys52Ter) and c.233G>A (p.Cys78Thr) in exon 2 and c.1010G>A (p.Cys337Thr) in exon 5 to account for 70.7% of the total number of mutations and 18.9, 21.5, and 30.3%, respectively, across 79 families from India (Bhavani et al., 2015). Although c.156C>A (p.Cys52Ter) is the most common recurrent variant worldwide, c.233G>A (p.Cys78Thr) and c.1010G>A (p.Cys337Thr) were observed to be the most common variants in the Indian population (Bhavani et al., 2015; Bhavani et al., 2020).

Interestingly, c.298T>A (p.Cys100Ser) variant, which was reported as a novel by Bhavani *et al.*, was observed in three separate families in our study. Furthermore, Bhavani *et al.* had also observed c.296A>T (p.Tyr99Phe) in *cis* with c.298T>A variant in their patient. In concordance with this observation, we also observed c.296A>T variant across all three patients (Figure 3) in our study, although in contrast, we could not find evidence to classify it as benign/tolerated based on *in silico* predictions from DANN (Quang et al., 2015), FATHMM-MKL (Shihab et al., 2015), PROVEAN (Choi and Chan, 2015), and MetaDome (Wiel et al., 2019). Determination of the functional effect of these variants would require functional studies, which is beyond the scope of the current study.

Across all three cases, we observed interphalangeal joint involvement, large joint involvement, spine involvement, and short stature (<third centile) as key clinical features (Table 1). Enlarged chest and square vertebrae were observed in cases 1 and 2 but not in case 3. Although families of cases 2 and 3 were lost to follow-up, the family of case 1 were subsequently invited for genetic counseling and treatment of phenotype manifestation. Following counseling, case 1 is treated by physical therapy to manage small joint arthropathy, although surgical interventions such as bracing for scoliosis were not carried out. Phenotypic assessment of parents of case 1 suggests no obvious clinical PPD phenotype, although interestingly, the father had telltale signs of enlargement of interphalangeal joints of hands (Figure 2K), which is in contrast to the asymptomatic obligate carriers observed in the literature. Although this is a noteworthy observation, the search for a cause of this phenotype in the father was beyond the scope of the study.

**TABLE 1:** Summary of the clinical and radiographic findings of all three cases.

	Clinical findings												Radiographic findings			
Case	Healthy at birth	Onset of arthropathy early in childhood, usually between ages three and 6 years	Enlargement of interphalangeal joints of hands	Progressive restricted mobility of all joints	Gait abnormalities	Genu valgum/genu varum	Progressive hip disease (commonly coxa vara at the late stage)	Articular pain	Motor weakness and fatigability	Spine involvement in late childhood and adolescence with thoracolumbar kyphoscoliosis that leads to short trunk	Adult height below the third centile	Absence of signs of inflammation	Spondyloepiphyseal dysplasia	Generalized arthropathy	Distinctive joint deformity of the hands	Diffuse osteoporosis
1	+	+	+	Uk	+	+	Uk	Uk	Uk	+	NA	+	+	+	+	Osteopenia
2	+	Uk	+	Uk	+	+	Uk	Uk	Uk	+	+	+	+	+	+	Uk
3	+	+	+	+	+	Uk	Uk	Uk	Uk	+	NA	+	+	+	+	Uk

+, present; -, absent; UK, unknown; NA, not applicable.

In concordance with the literature, we observe a higher than anticipated number of people affected with PPD in the Patni community of Gujarat. This community lives across the western part of India and Sindh province in Pakistan and is known to practice endogamy frequently. This could explain the observation of the same causative variant across three independent families from this community. Therefore, this suggests the utility of testing c.298T>A variant first in a case suspected with PPD belonging to the Patni community.

## Conclusion

We reported three PPD cases of individuals from three independent families belonging to the Patni community of Gujarat who were homozygous for a pathogenic variant c.298T>A in the *CCN6* gene. The literature review suggests this to be the first instance from India with a recurrent variant being detected in the *CCN6* gene in patients belonging to an endogamous community. In summary, our study suggests c.298T>A in *CCN6* gene to be a recurrent variant in patients clinically suspected with PPD belonging to the Patni community.

## Data Availability

The datasets for this article are not publicly available due to concerns regarding participant/patient anonymity. Requests to access the datasets should be directed to the corresponding author.
